# Rotation-tolerant representations elucidate the time-course of high-level object processing

**DOI:** 10.1371/journal.pone.0347992

**Published:** 2026-04-29

**Authors:** Denise Moerel, Tijl Grootswagers, Amanda K. Robinson, Patrick Engeler, Alex O. Holcombe, Thomas A. Carlson

**Affiliations:** 1 School of Psychology, University of Sydney, Sydney, Australia; 2 The MARCS Institute for Brain, Behaviour and Development, Western Sydney University, Sydney, Australia; 3 School of Computer, Data and Mathematical Sciences, Western Sydney University, Sydney, Australia; 4 Queensland Brain Institute, The University of Queensland, Brisbane, Australia; 5 School of Psychology, the University of Queensland, Brisbane, Australia; Kochi University of Technology, JAPAN

## Abstract

Despite the very different retinal images that result from different viewing conditions, humans have little difficulty recognising visual objects in varying circumstances. One source of variability is 2-D rotation, which results in an object having different orientations. Here, we studied how the brain transforms rotated object images into object representations that are tolerant to rotation. We measured time-varying electroencephalography responses to object images shown in eight different orientations, presented at either 5 Hz or 20 Hz. We used multivariate classification to assess when rotation-tolerant object information emerged, and whether the rotation-tolerant processing would be limited at the faster presentation rate. We compared this to fixed-rotation measures of object decoding, where the classifier is trained and tested on the same orientation. Our results showed that both fixed-rotation and rotation-tolerant object decoding emerged at an early stage of processing, less than 100 ms after stimulus onset. However, rotation-tolerant information peaked later than fixed-rotation information, suggesting rotation-tolerant object representations are most prominent during a late stage of processing, around 200 ms after stimulus onset. Both fixed-rotation and rotation-tolerant object information was lower for the 20 Hz compared to 5 Hz presentation rate, which suggests that object information processing is disrupted, but not eliminated, for fast presentation rates. Our results show that object information arises at similar times in the brain regardless of whether it is investigated with the fixed-rotation or rotation-tolerant object decoding method. An object representation that is tolerant to rotation and generalises across different exemplars of the same object is established in later stages of processing.

## 1. Introduction

Humans have little difficulty recognising objects across changes in rotation, size, position, and lighting. This implies that the brain creates and maintains representations of objects that are robust to these changes [1,2]. Robust object representations can provide efficient neural encoding by reducing multiple specific object representations (e.g., different rotations) into one. This suggests that a given image elicits image-specific neural responses based on low-level features and is then transformed into a general object representation. Previous work has used multivariate decoding methods to provide insight into the dynamics of visual object representations in the human brain [3–6]. However, these studies have not assessed whether the decoded object information from the brain is driven by image-specific low-level features or whether it represents an object representation that is invariant, or tolerant, to transformations such as rotation. Here, we use the term ‘tolerant’ [2,7,8] to highlight the different possible degrees of transformation tolerance, as opposed to binary ‘invariance’. The present study was designed to yield insights into how objects are represented in the human brain and the process by which this specific to general transformation occurs. To this end, we compare the time-course of image-specific and rotation-tolerant representations.

Non-human primate studies have shown that object representations that are tolerant to different transformations emerge in the ventral visual stream. For instance, single neurons in the inferior temporal cortex (IT) are tolerant to rotation [9] as well as size and position [8,10–12] and image contrast [8], whereas neurons in earlier visual areas such as primary visual cortex respond to image specific features [13]. In addition, tolerance to image transformations increases as information propagates along the ventral visual stream from V4 to IT [2]. A non-human primate study found that size-tolerance developed early after stimulus onset, followed by position-tolerant signals, and then by rotation-tolerant and view-tolerant signals [14]. The studies from non-human primates provide evidence for the emergence of transformation-tolerant object representations as information propagates along the ventral visual stream.

It is important to distinguish between rotation-tolerance and view-tolerance. Rotation-tolerance refers to rotations in the picture plane, which do not reveal new object features, whereas view-tolerance refers to rotations that have a depth component, such that different aspects of the object become (in)visible. In this study, we focus on rotation-tolerance as a form of tolerance that the visual system can, in principle, achieve without relying on knowledge of the 3D structure of the object, similar to size or position tolerance. By studying rotation-tolerance, we gain insight into how the brain achieves robust visual recognition.

Previous decoding work with human participants has used magnetoencephalography (MEG) and electroencephalography (EEG) to investigate the time-course of the emergence of object information in the brain [3–6]. Specifically, the studies looked at three different levels of categorical abstraction: the “animacy level” (judgments of whether an image represented something that can move on its own volition), high-level categories (e.g., mammal, fruit, tool, etc.), and the object category level (e.g., giraffe, apple, hammer, etc.). The results showed evidence for object-specific information in the brain earlier than 100 ms after stimulus onset in each of these studies. However, it is still unclear whether this information reflects a representation that is tolerant to transformation. A linear classifier will use all information that helps to distinguish two objects from the neural signal, so the decoded object information is likely to be driven both by low-level differences between objects as well as higher-level object representations. This means that decoding times found in previous work might not be a good reflection of when the brain has truly established a representation of the object that is tolerant to transformation.

Studies that have investigated the emergence of high-level category information have found a different temporal signature compared to object exemplar decoding [3–6]. In these studies, the first decoding peak for object exemplar decoding is around 120 ms, but this peak is not usually observed in high-level category decoding. Instead, the high-level category decoding peak is found around 200 ms after stimulus onset. Information about animacy peaks later, usually around 400 ms after stimulus onset. This suggests that at 200 ms, a representation of the high-level object category has emerged and information from a specific exemplar is generalised to other exemplars within that category. This time-course is also consistent with object decoding when the training and test set contain different exemplars of the same object [3–6], again suggesting that the information has generalised to other exemplars within the category. It is possible that the time-course of the emergence of rotation-tolerant object representations resembles that of high-level category decoding, as both access representations that are more tolerant to low-level pixel-wise differences between stimulus images.

A few studies have used time-resolved neuroimaging to provide insight into how transformation tolerant object representations emerge throughout the human visual system. Using MEG, Carlson and colleagues [15] showed position-tolerant object representations emerged around 100 ms after stimulus onset. Isik and colleagues [16] used MEG to investigate the emergence of position- and size-tolerant information in object processing and found that tolerant representations emerged later than non-tolerant information. Size-tolerant information peaked first, with a later peak for position-tolerant information. In addition, tolerance to size and position increased along the ventral stream. Supporting this finding, EEG evidence showed tolerance to viewpoint followed size- and position-tolerance [17]. These findings from humans are in line with the findings in non-human primates reviewed above [14]. Together, they suggest that transformation-tolerant representations emerge while undergoing additional processing stages within the ventral visual stream. The studies described above have investigated the tolerance to different transformations but have not explored the tolerance to rotation specifically. It is therefore still unclear how *rotation-tolerant* object representations emerge in the human brain. Rotation is arguably a more complex transformation than the scaling and translation needed to reconcile size- and position-tolerance, which may be why rotation tolerance emerges later in non-human primates [14].

To investigate how the visual system reconciles different rotations into a robust representation, we investigated the time-course of rotation-tolerant information processing in the human brain in this study. In addition, we asked whether we could disrupt the rotation-tolerant object processing by presenting stimuli at faster speeds, by comparing 5 Hz and 20 Hz presentation rates. The 5 Hz condition provides 200 ms of uninterrupted processing, which allows recurrent processes to contribute more strongly to the resulting representations, and visual information is therefore expected to reach high levels of processing [18]. In contrast, the 20 Hz condition only provides 50 ms of uninterrupted processing, with processing likely to be dominated by feedforward mechanisms, biasing visual representations to earlier processing stages. In line with this, previous work has shown that faster presentation rates limit visual information processing [6,19–22], particularly high-level information processing [21,23]. We presented sequences of object stimuli in eight in-plane rotations, at 5 Hz and at 20 Hz, and used multivariate decoding methods to investigate the coding of rotation-tolerant information over time. The comparison between rotation-tolerant and fixed-rotation object decoding can show how the brain transforms image-specific representations into a general object representation.

## 2. Methods

### 2.1. Participants

Sixteen healthy adults participated in the study (age range = 19–60 years, 14 female/ 2 male, 14 right-handed/ 2 left-handed). All but one participant reported having normal or corrected to normal vision, with one participant reporting a history of strabismus. We therefore conducted a visual inspection of this participant’s data for the fixed-rotation and rotation-tolerant decoding analyses for the 5 Hz presentation condition (Fig 3A). The data did not exhibit any notable deviations relative to the rest of the sample, and the participant was retained in the group-level analyses. Participants received monetary payment or undergraduate course credit for their participation. The participants were recruited between 26/2/2020 and 13/3/2020. The study was approved by the ethics committee of The University of Sydney, and participants provided both written and oral informed consent.

### 2.2. Stimuli and experiment procedure

**Fig 1:**
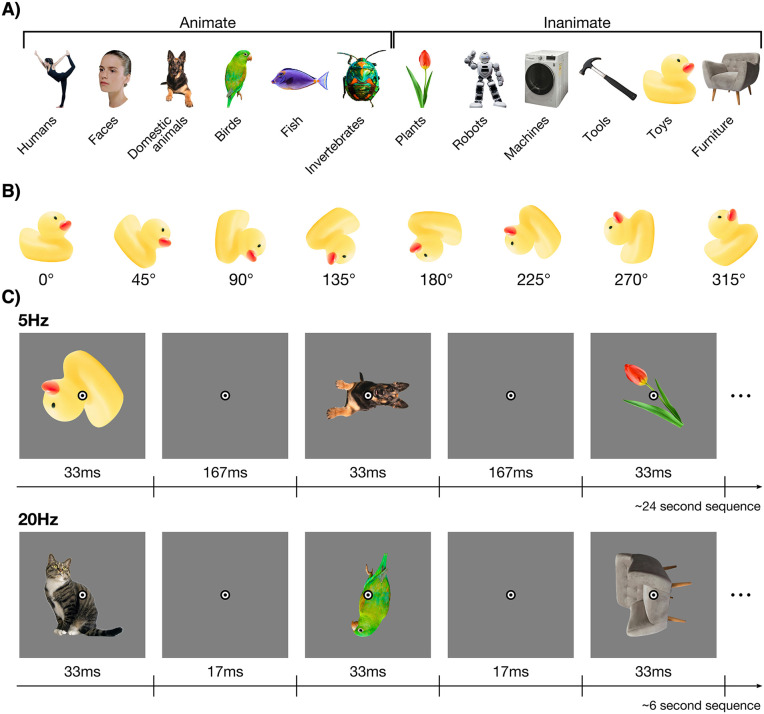
Stimuli and experimental design. Some stimuli are replaced with visually similar images from the public domain. **A)** Example stimuli. There were 60 stimuli in total, grouped into two categorical levels: 1) animacy, and 2) object category. Each object category contained five exemplars. The 0° rotation is displayed in **A. B)** The eight rotations that each stimulus was presented in ranged from 0° to 315° in steps of 45°. **C)** The stimulus sequences were presented at two frequencies: 5 Hz and 20 Hz. The stimuli were presented for 33 ms in both presentation frequency conditions, so the interval between consecutive stimuli was 167 ms for the 5 Hz condition and 17 ms for the 20 Hz condition.

Fig 1A provides an overview of the stimuli. The stimulus set consisted of 60 objects, taken from Contini et al. [24] and an online image bank (www.pngimg.com). The images can be grouped at the animacy level, with 30 animate and 30 inanimate images, or at the category level, with 12 categories (e.g., humans, birds, toys). Each category contained five objects. We presented the object stimuli in eight rotations: 0°, 45°, 90°, 135°, 180°, 225°, 270°, and 315° (see Fig 1B). The stimuli were presented at fixation on a mid-grey background (RGB = 128,128,128), at a distance of approximately 60 cm. The stimuli were 512 × 512 pixels (approximately 12.50 degrees visual angle) in size.

Participants viewed 120 sequences of images, and each sequence contained 120 object images. There were 15 repetitions per object, rotation, and presentation frequency condition, resulting in a total of 14,400 stimulus presentations. In each sequence the 60 images were shown twice in two different rotations. The stimulus items in each sequence were presented in random order, and each object-rotation combination in the sequence was unique compared to the other stimuli in the sequence. Four consecutive sequences contained a single repeat of all objects and rotations for a single presentation duration, and the presentation duration was therefore constant across four consecutive sequences. We presented the image sequences at two frequencies, 5 Hz and 20 Hz. Fig 1C shows part of an example sequence for both presentation frequency conditions. Stimuli were presented for 33 ms in both presentation frequency conditions. In the 5 Hz condition, the interval between successive stimuli was 167 ms, whereas the interval was 17 ms in the 20 Hz condition. Using a periodic, rather than jittered, stimulus presentation was chosen as previous work showed periodicity does not influence processing in the first 300 ms [25], and this allowed a more direct comparison with previous work using the same presentation frequencies [6,20,22]. Each sequence had a single stimulus presentation frequency, and the counterbalanced presentation frequencies were presented in pseudo-random order. The experiment took approximately 45 minutes to complete, and participants were given the opportunity to take a break after every sequence.

Participants were instructed to maintain fixation at a central bullseye for the duration of the experiment and to respond to an infrequent target by pressing a button. This task was orthogonal to the objects, and the objects were therefore not task-relevant. The purpose of this task was to keep participants engaged in the experiment, and to ensure participants remained fixating at the centre of the objects. The bullseye consisted of two black rings and a white ring in between (see Fig 1C). The target consisted of a black filled circle the same size as the fixation bullseye and was presented instead of the fixation bullseye for the duration of a stimulus presentation (33 ms). Each sequence contained between two and four targets. We placed the targets at random in each sequence, using the following constraints. Two targets were spaced at least 10 stimulus presentations apart, and the first and last 10 stimulus presentations were never a target. We counted responses within 1 s of the target as correct. Participants had an average accuracy of 88.7% on the fixation colour change detection task (SEM = 2.1%, range = 70.5% to 99.7%), 94.6% for the 5 Hz condition (SEM = 1.3%, range = 79.0% to 100.0%) and 82.9% for the 20 Hz condition (SEM = 3.7%, range = 51.4% to 99.5%).

### 2.3. EEG acquisition and pre-processing

We used a BrainVision ActiChamp system to record continuous EEG data from 128 electrodes, digitised at a sample rate of 1000 Hz. The electrodes were placed according to the international standard 10–20 electrode placement system [26], and were referenced online to FCz. We used the EEGlab Matlab toolbox to pre-process the data [27], following a pre-processing pipeline used in previous work [28,29]. We interpolated bad channels that measured more than 5 standard deviations away from the average, using the kurtosis measure. We re-referenced the data to an average reference and filtered the data using 0.1 Hz high pass and 100 Hz low pass Hamming windowed FIR filters. We down-sampled the data to 250 Hz and created epochs for each stimulus in the sequence from −100 ms to 800 ms relative to stimulus onset.

### 2.4. Decoding analysis

We applied decoding analyses to determine the time-course of fixed-rotation measures of object information and rotation-tolerant object information, using the CoSMoMVPA toolbox for Matlab [30]. We performed the analyses within-subject, separately for the 5 Hz and 20 Hz sequences. For each time-point in the epoch, we trained a linear discriminant analysis (LDA) classifier to distinguish between the 60 objects (see Fig 1A). As we used a 60-way object classification analysis, chance performance was 1.67%. To decode the fixed-rotation object information, we followed the method used in previous work [3–6], by training the classifier on the same rotation as used in the test set. For each unique combination of object and rotation, there were 15 repetitions of the identical stimulus over the entire experiment for each presentation speed condition. The training set consisted of 14 of the 15 identical stimulus repetitions of each of the 60 objects and the test set consisted of the left-out stimulus repetition of each object in the same rotation, for example training on 14 repetitions of all 60 objects at 0° rotation and testing on one repetition of all 60 objects at 0°. We repeated this analysis 15 times, leaving out a different stimulus repetition of each of the 60 objects each time, and averaged across decoding accuracies. We also repeated this analysis for all the rotations, and averaged decoding accuracies across them. This analysis was done for each time-point. To decode rotation-tolerant object information, we trained the classifier to distinguish between the 60 objects on 7 different rotations of an object and tested the classifier on the eighth rotation. We ensured that the classifiers were trained and tested on the same amount of data as the fixed-rotation method by always training on 14 repetitions of each object and testing on one repetition of each object, for example training on 14 repetitions of all 60 objects, with 2 repeats per orientation (45°, 90°, 135°, 180°, 225°, 270°, and 315°) and testing on the left-out repetition of all 60 objects at 0°. Note that we tested the classifier on the exact same trials for the fixed-rotation and the rotation-tolerant object decoding analyses. The only difference between the different analyses was that the models were always trained and tested on the same rotation angle in the fixed-rotation object decoding analysis, consistent with previous studies [3–6], whereas rotation-tolerant models were trained and tested on different rotation angles. It is therefore possible to directly compare classifier accuracies between fixed-rotation and rotation-tolerant object decoding analyses.

### 2.5. Exploratory analyses

#### 2.5.1. Channel searchlight.

To investigate which EEG channels were driving the classification accuracies, we obtained time-varying topographies by performing an exploratory channel-searchlight analysis, following an established pipeline [20,22]. We constructed a local cluster for each EEG channel by taking the closest 4 neighbouring channels and ran the decoding analysis described above separately for the fixed-rotation and rotation-tolerant object coding analyses, for the 5 Hz and 20 Hz conditions. The decoding accuracy for each cluster was stored in the centre channel, resulting in a time-by-channel map of the decoding accuracies for each condition, separately for each participant.

#### 2.5.2. Representational similarity analysis.

**Fig 2:**
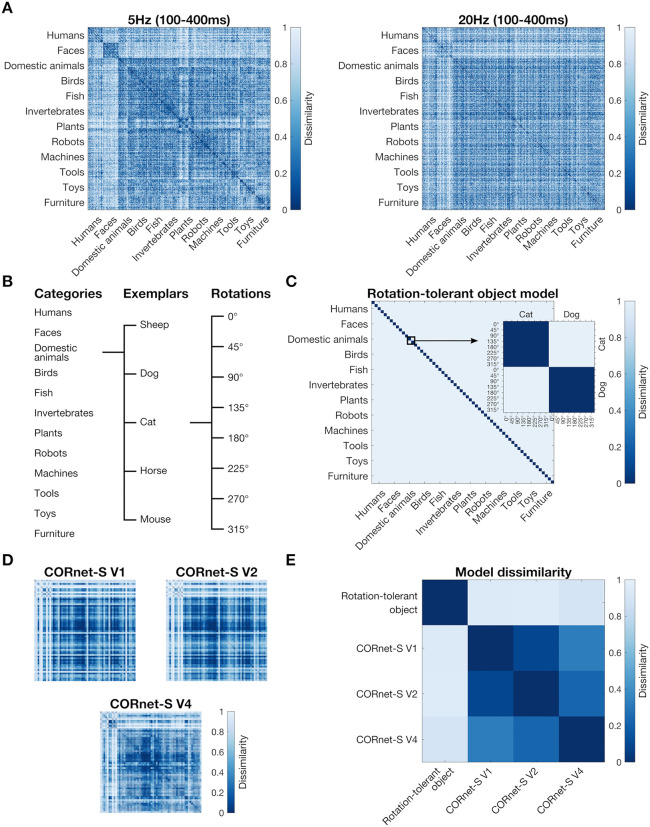
Models used in the Representational Similarity Analysis. **A)** The representational dissimilarity matrix (RDM) for the EEG data, averaged across the 100 ms to 400 ms time-window, for the 5 Hz (left) and the 20 Hz (right) condition. Each point in the 480 by 480 matrix represents the dissimilarity between two stimuli. **B)** The labels for all 480 by 480 matrices plotted here: the rows and columns consist of 12 categories × 5 exemplars × 8 rotations. **C)** The rotation-tolerant object model. This model predicts that the response to the same object is very similar, regardless of the orientation in which the object is presented, whereas the response to two different objects is dissimilar. **D)** The CORnet-S control models that were used to control for low-level visual differences between the stimuli. **E)** The dissimilarity between the four different models: the rotation-tolerant object model and the three control models.

We used the Representational Similarity Analysis (RSA) framework [31,32] to investigate the temporal dynamics of rotation-tolerant object representations, while reducing the contribution of low-level differences in image features. This framework allows us to compare the representational structure obtained by EEG to different models of object representations, using representational dissimilarity matrices (RDMs) that represent the dissimilarity between item activations. Fig 2 gives an overview of this analysis. We created a 480 by 480 neural RDM from the EEG data, which coded for the neural dissimilarity between each combination of unique stimuli (60 object images in 8 rotations, see Fig 2B). We used cross-validated pairwise decoding accuracy as a measure of dissimilarity, as a more dissimilar neural response between items results in a higher decoding accuracy. We obtained a neural RDM for each participant, separately for each time-point and for the 5 Hz and 20 Hz stimulus presentation conditions (Fig 2A). For each time-point, we correlated the lower triangle of the neural RDM to a model RDM that codes for the object in a rotation-tolerant way (Fig 2C).

We partialled out three control models from this correlation (Fig 2D), to control for low-level differences in image features, following previous work [33,34]. We used the layers “V1”, “V2”, and “V4”, from CORnet-S [35], a deep convolutional artificial neural network, as control models. These layers are designed to be analogous to the ventral visual areas V1, V2, and V4 respectively. We used cosine as a distance measure to obtain the dissimilarity between each pair in the 480 by 480 RDM. The dissimilarities between the three CORnet control models and the rotation-tolerant object model are shown in Fig 2E.

#### 2.5.3. Temporal generalisation.

We applied the temporal generalisation approach [15,36,37] to determine 1) whether object representations at one time-point generalised to other times, and 2) whether the object representations were similar for the 5 Hz and 20 Hz presentation conditions. Instead of training and testing the classifier on a single time-point, the classifier is trained and tested on all combinations of time-points. This results in a training time by test time time-generalisation matrix and allows us to capture similar object representations that occur at different times. To investigate the temporal generalisation between object representations over time, we trained and tested a classifier in the same way as described in the decoding analysis above for each combination of training and testing time-points. We did this separately for the 5 Hz and 20 Hz conditions. To determine the overlap in object representations between the 5 Hz and 20 Hz presentation conditions, we trained a classifier on the 5 Hz condition for a time-point in the epoch and then tested the classifier on every time-point in the 20 Hz condition. We repeated this analysis for all training time-points. In addition, we trained on the 20 Hz condition and tested on the 5 Hz condition, and averaged the two time-generalisation matrices [6,38].

### 2.6. Statistical inference

We used Bayesian statistics [39–43] to determine whether decoding accuracies were at chance level, or instead substantially higher. We used the Bayes Factor R package [44] to implement the statistical analyses. We implemented the same analysis for the fixed-rotation and the rotation-tolerant object decoding analyses, for both the 5 Hz and the 20 Hz presentation conditions. For each time-point, we applied a Bayesian t-test with a point null and a notched half-Cauchy prior for the alternative hypothesis to test for directional effects [45]. The half-Cauchy prior was centred around d = 0 (i.e., chance-level decoding accuracy of 1.67%) and had the default width of 0.707 [42,46,47]. We excluded the interval between d = 0 and d = 0.5 (the “notch”) from the prior [48]. Because this analysis pits a point null hypothesis against a d > 0.5 alternative hypothesis, this analysis tests whether the decoding is substantially above chance not just marginally above chance. We repeated this analysis for each time-point. All statistical analyses were applied at the group level.

To test whether there was a difference between the coding of object information found with the fixed-rotation and rotation-tolerant object decoding analyses, we calculated the difference between these decoding accuracies and used a Bayesian t-test to test the difference score against 0 for each time-point. We used a point null and a full-Cauchy prior for the alternative hypothesis to test for differences in either direction between the coding of object information found with the fixed-rotation and rotation-tolerant object decoding analyses. For consistency with the t-test described above, we used the default prior width of 0.707 with a notch (excluding the interval between d = −0.5 and d = 0.5) from the prior.

We used the same statistical analysis described above for the representational similarity analysis. For the temporal generalisation analysis, we repeated the same statistical analysis for each combination of training and test time-points.

## 3. Results

### 3.1. Fixed-rotation and rotation-tolerant object decoding analyses

**Fig 3:**
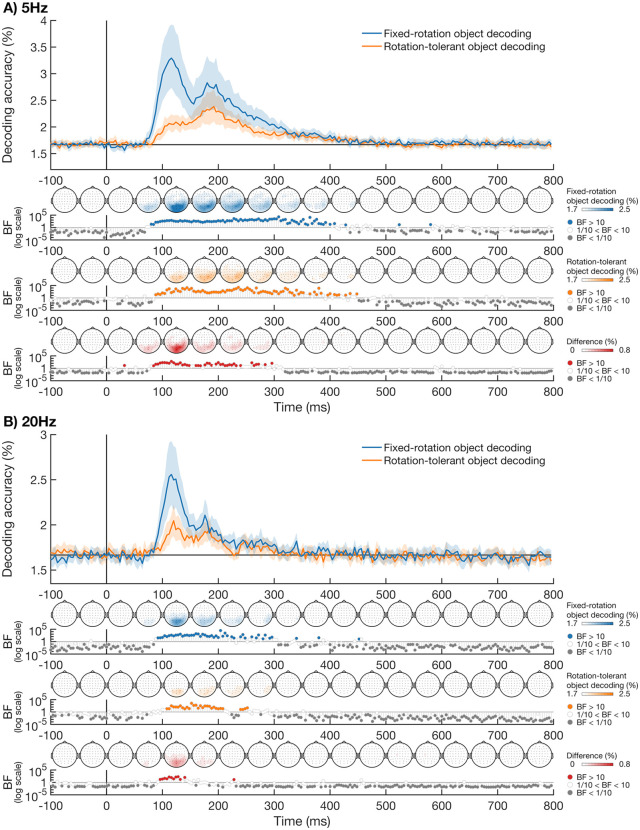
Time-course of object decoding accuracy for 5 Hz (A) and 20 Hz (B) presentation. **A)** Object decoding data for the 5 Hz presentation. We used linear classifiers to decode object information via the fixed-rotation decoding method (blue), that were trained and tested on the same rotation, and in a rotation-tolerant way (orange), where we trained and tested on different object rotations. Decoding accuracies are based on 60-way decoding, which means chance is 1.67% decoding accuracy. The shaded areas around the plot lines show the bootstrapped 95% confidence intervals across participants. The time-varying topographies, which were obtained from the exploratory channel-searchlight analysis, are displayed below each plot, averaged within 50 ms time bins. We obtained separate topographies for the fixed-rotation (blue) and rotation-tolerant (orange) object decoding methods. Notched Bayes factors (excluding small positive effect sizes from the prior) are shown below the plot. Notched Bayes factors below 1/10 are shown in grey and those above 10 are shown in colour. **B)** Object decoding accuracy for the fixed-rotation analysis (blue) and the rotation-tolerant (orange) analysis for the 20 Hz presentation condition. Plotting conventions are the same as in **A.**

The temporal dynamics of object representations obtained with fixed-rotation decoding and a rotation-tolerant decoding method are shown in Fig 3. For the 5 Hz presentation condition (Fig 3A), there was clear evidence (BF > 10) for substantially above-chance fixed-rotation object decoding from ~84 ms after stimulus onset. Clear evidence for substantially above chance decoding of the rotation-tolerant object information emerged at a similar time, approximately 92 ms after stimulus onset. Visual inspection shows there are two distinct peaks, between approximately 100 ms to 150 ms after stimulus onset, and between approximately 150 ms to 250 ms after stimulus onset. For the object coding found through the fixed-rotation method, the first peak was higher compared to the second peak, with the overall peak found at ~116 ms after stimulus onset. In contrast, the second peak was higher than the first for the rotation-tolerant decoding, with the overall peak found at ~192 ms after stimulus onset. From ~88 ms after stimulus onset, the fixed-rotation object decoding had higher accuracy than the rotation-tolerant object decoding. Together, these results suggest that object information emerges around a similar time regardless of whether we trained and tested on the same orientation or on different orientations. However, rotation-tolerant object information peaked later than the fixed-rotation analysis, suggesting that object representations are most tolerant to image rotation at later stages of processing.

We repeated the same analysis for the 20 Hz presentation condition. We aimed to provide insight into the depth of object processing by comparing object coding for fast and slower presentation rates. Previous work showed a decrease in object decoding accuracy when stimuli are presented at a fast compared to slower presentation rate [22], suggesting that faster presentation rates impaired visual processing. Fig 3B shows the decoding accuracies for object coding for the 20 Hz presentation condition, obtained for the fixed-rotation and rotation-tolerant analyses. Both fixed-rotation and rotation-tolerant object information could be decoded at a similar time. For fixed-rotation decoding, when the classifier was trained and tested on the same rotation, the evidence began to clearly favour substantially above-chance decoding from ~96 ms after stimulus onset, with peak decoding at ~116 ms. There was clear evidence for substantially above-chance rotation-tolerant object decoding from ~112 ms after stimulus onset, peaking at ~120 ms after stimulus onset. There was substantial evidence for stronger object decoding for the fixed-rotation analysis compared to the rotation-tolerant analysis starting at ~100 ms after stimulus onset. These results show that rotation-tolerant information still emerges for a high presentation rate, though there was not an obvious late peak as in the slower condition.

Directly comparing the 5 Hz and 20 Hz presentation conditions with a notched Bayesian t-test showed object coding was stronger for the 5 Hz condition for both the fixed-rotation analysis, starting at ~92 ms after stimulus onset, and the rotation-tolerant analysis, starting at ~104 ms after stimulus onset. Together, these results suggest that the 20 Hz presentation speed limits, but does not eliminate, both fixed-rotation and rotation-tolerant object processing.

### 3.2. Representational similarity analysis

In the analysis described above, we assessed the decoding accuracy for rotation-tolerant object information by training the classifier on all but one rotation and testing the classifier on the left-out rotation. This means that successful rotation-tolerant object decoding requires generalisation between different rotations and cannot depend on specific pixel-wise configurations between stimulus images. This decoding analysis is therefore less likely to reflect low-level differences in object features compared to the fixed-rotation object decoding. However, it is possible that the decoding of the rotation-tolerant object is driven in part by low-level visual differences between the object images. We used representational similarity analysis to account for low-level visual differences, by using control models based on CORnet-S. This allows us to investigate the time-course of rotation-tolerant object coding when further reducing the contribution of these low-level visual differences.

**Fig 4:**
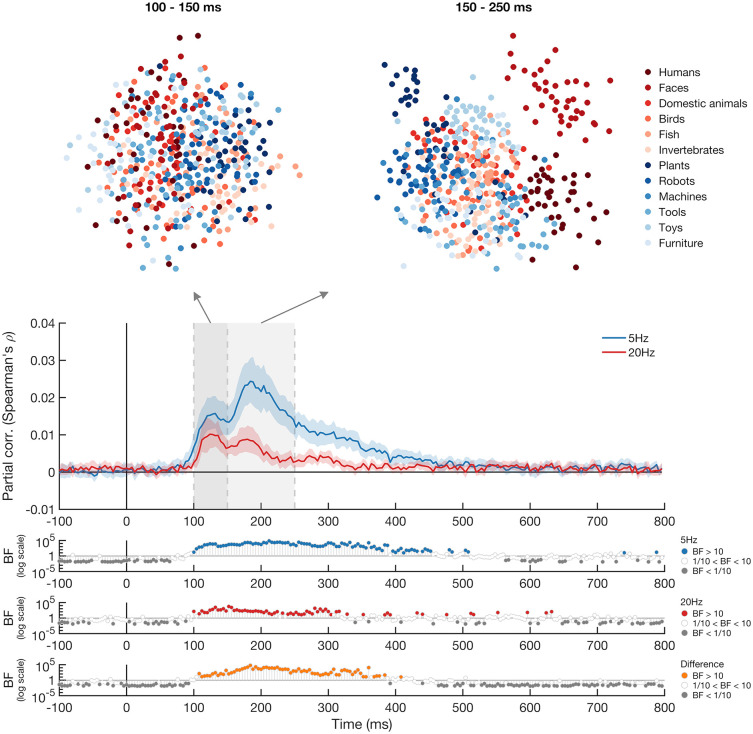
Representation Similarity Analysis results. The blue and red lines show the partial correlation of the rotation-tolerant object model with the EEG data for the 5 Hz condition (blue) and the 20 Hz condition (red) over time, with the three visual control models partialled out. The bootstrapped 95% confidence intervals across participants are displayed as shaded areas around the plot lines. Notched Bayes factors are shown below the plot, with Bayes factors below 1/10 displayed in grey and Bayes factors above 10 shown in the plot colour. Above the plot are projections of the neural dissimilarity of the stimuli (presented at 5 Hz) arranged in a two-dimensional space. The left plot is based on the EEG data between 100 ms and 150 ms after stimulus onset, roughly corresponding to the first peak of the partial correlation of the rotation-tolerant object model with the EEG data for the 5 Hz condition. The right plot is based on the EEG data between 150 ms and 250 ms after stimulus onset, roughly corresponding to the second peak. The distance between two stimuli reflects their pairwise distance, with larger distance between two stimuli reflecting more dissimilar neural responses. Each dot represents a stimulus and the colours represent the 10 different categories. The categories become noticeably more separated at the later time-window compared to the earlier time-window, especially the people and faces.

Fig 4 shows the partial correlation between the EEG data and the rotation-tolerant model for each presentation speed condition after accounting for the three control models. For the 5 Hz presentation condition, substantial evidence for a partial correlation with the rotation-tolerant object representation model was present from ~104 ms after stimulus onset. The peak time of this partial correlation was ~ 184 ms after stimulus onset. The timings for the 20 Hz condition were very similar, we found substantial evidence for a partial correlation with the rotation-tolerant object model from ~112 ms after stimulus onset. The time-courses for the representational similarity analysis are very similar to those found in the decoding analysis, suggesting rotation-tolerant object representations are evident when images are presented at both 5 Hz and 20 Hz and are unlikely to be driven by low-level visual information about the different objects. Directly comparing the partial correlations of the rotation-tolerant object model with the EEG data from the 5 Hz and 20 Hz conditions, a difference was evident from ~112 ms after stimulus onset, with a higher correlation with the rotation-tolerant object model for the 5 Hz compared to 20 Hz condition.

We made a two-dimensional embedding of the EEG RDM to visualise the dynamic representational structure for the images presented at 5 Hz, during the first peak (100 ms – 150 ms) and second peak (150 ms – 250 ms) (Fig 4, top panel). The distance between the images reflects their mean dissimilarity across participants. At 150 ms – 250 ms after stimulus onset, a clear clustering emerges of different rotations of the same images, suggesting the object representation is then tolerant to rotation. In addition, the faces and bodies become separated from the other object categories at this time, suggesting there is a distinct representation of human bodies and human faces at 150 ms – 250 ms after stimulus onset.

### 3.3. Temporal generalisation

**Fig 5:**
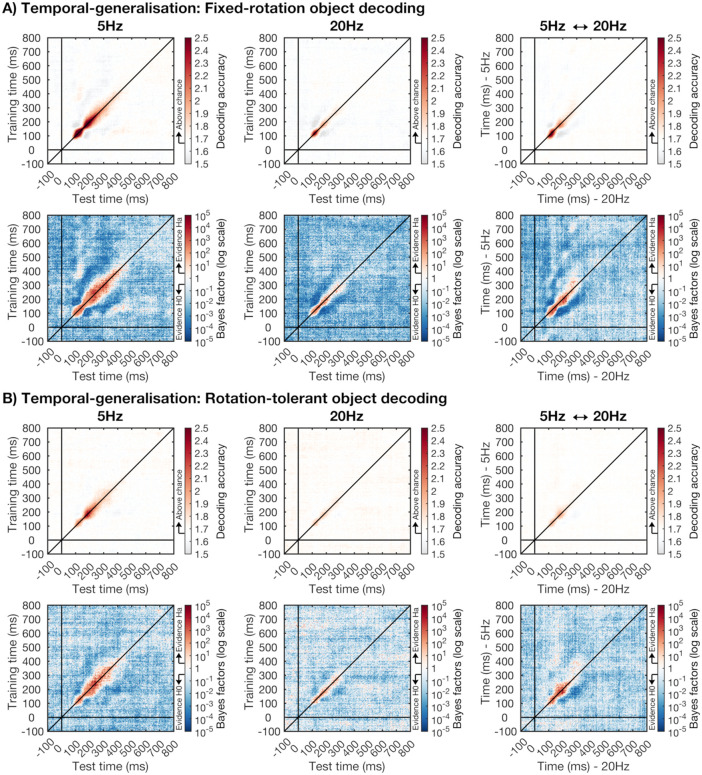
Temporal-generalisation for the fixed-rotation object decoding (A) and rotation-tolerant object decoding (B). We used 60-way object decoding, which means that theoretical chance is at 1.67%. **A)** Shows the temporal-generalisation of decoding accuracies for the fixed-rotation object coding analysis (top row), with the corresponding notched Bayes factors on a logarithmic scale (bottom row). The left panels show the results for the 5 Hz stimulus presentation condition, and the middle panels for the 20 Hz presentation condition. The training times are shown on the y-axis and the test times on the x-axis. The right panels show the cross-decoding between the 5 Hz and 20 Hz condition. For this analysis, we trained on the 5 Hz condition and tested on the 20 Hz condition and vice versa. We averaged the results across the two analyses, transposing the train on 5 Hz test on 20 Hz matrix. The y-axis shows the times for the 5 Hz condition, and the x-axis the times for the 20 Hz condition **B)** shows the temporal-generalisation of decoding accuracies for the rotation-tolerant object coding (top row) and the corresponding notched Bayes factors on a logarithmic scale (bottom row). Plotting conventions are the same as in **A.**

We used the temporal generalisation approach to investigate 1) whether activation patterns observed at one time-point generalise to other time-points, and 2) whether the object representations were similar between the 5 Hz and 20 Hz presentation conditions. Temporal generalisation results are displayed in Fig 5. To determine whether activation patterns generalise between different time-windows, we trained and tested the classifier to distinguish between objects for all combinations of training and test time-points. We did this separately for the fixed-rotation (Fig 5A) and rotation-tolerant (Fig 5B) object decoding analyses, and for the 5 Hz and 20 Hz presentation conditions. Decoding was strongest along the diagonal for all conditions, when the classifier was trained and tested on time-points that were close in time. While some generalisation was present for neighbouring time-points, the Bayes factors suggest no generalisation between the two different decoding peaks, around 120 ms and 200 ms, which suggests that the decoding in these two time-windows is driven by different patterns of activation. However, there was some evidence for generalisation between the first decoding peak and a much later time-window, around 350 ms to 500 ms after stimulus onset.

To determine whether the object representations were similar for the two different presentation rates, we trained on the 5 Hz condition and tested on the 20 Hz condition and vice versa and collapsed the time-generalisation matrices for these two analyses. We transposed the train on 20 Hz, test on 5 Hz time-generalisation matrix before averaging, which means above-chance decoding off the diagonal shows that similar activation occurs at different times for the two presentation speed conditions. We found evidence for cross-decoding between the 5 Hz and 20 Hz conditions for both the fixed-rotation and the rotation-tolerant object decoding analyses. This suggests that the object representations were similar for the two presentation rates. In addition, the decoding was primarily along the diagonal for the cross-decoding between the 5 Hz and 20 Hz conditions, indicating this information was processed around the same times in both speed conditions. However, there was stronger decoding above compared to below the diagonal for the rotation-tolerant object decoding, suggesting the rotation-tolerant object coding for the 5 Hz condition lasted longer compared to the 20 Hz condition.

## 4. Discussion

In this study we investigated the time-course of neural information about object representations that are tolerant to rotation. In line with previous studies [3–6], our results showed evidence for object coding, including above-chance decoding for the object exemplar when we used the fixed-rotation method of training and testing on the same object rotation. Our results add to these findings by providing evidence for the coding of *rotation-tolerant* object information, and directly contrasting object decoding found through the fixed-rotation decoding method and the rotation-tolerant object decoding method. The evidence began to favour substantially above-chance object decoding around similar times for the fixed-rotation and rotation-tolerant decoding methods, approximately 84–92 ms after stimulus onset. However, the decoding of the rotation-tolerant object information peaked later than the decoding accuracy obtained via the fixed-rotation method, suggesting that rotation-specific information is transformed into a higher-level representation, tolerant to rotation, at later stages of processing. When we presented object stimuli at a faster rate (20 Hz), there was still evidence of both fixed-rotation and rotation-tolerant neural representations, although decoding accuracies were lower for stimuli presented at the faster compared to slower (5 Hz) presentation rate.

Previous work has investigated the time-course of object representations that are tolerant to size and position [16]. The authors found that standard object coding, that was not necessarily tolerant to size or position, peaked at 135 ms. Size-tolerant decoding peaked at 170 ms, and position-tolerant decoding peaked at 180 ms after stimulus onset. Our findings add to this by investigating rotation-tolerant decoding. We showed that fixed-rotation object coding peaked ~116 ms after stimulus onset, whereas rotation-tolerant object coding peaked at ~192 ms after stimulus onset. Although it is not possible to directly compare the peak times across these different studies because of different stimuli and methods, our findings suggest that the rotation-tolerant representation is fully established later than the size-tolerant and position-tolerant representation. This would be in line with findings in non-human primates [14] as well as findings in humans at the category level [17]. Our findings therefore contribute to our understanding of how the human brain arrives at object representations that are tolerant to different transformations. In addition, our RSA method adds to previous findings by controlling for the contribution of low-level visual differences between stimuli. An object presented in different sizes, positions, or rotations will have consistent low-level visual features, such as luminance, which can be used by a classifier. The RSA method used in this study controls for the contribution of these features, allowing us to focus on high-level representations that are tolerant to differences in rotation.

Previous work has provided insight into the time-course of the emergence of object information in the brain using fixed-rotation decoding methods, where the classifier is trained and tested on objects presented in the same (canonical) orientation [3–6]. One limitation of this method is that differences in activation patterns caused by the different objects can be driven by low-level differences between objects. It is possible that there is information in perceptual areas about the low-level differences between objects, without having true object representations. The classifier cannot distinguish between these two possibilities and is likely to rely on both. By training and testing the classifier on different object rotations, we can assess the object-related information that generalises between different rotations. This helps us tap into the higher-level representations of objects that are tolerant to rotation, minimising the contribution of low-level differences in object features. Our results show a peak in rotation-tolerant object coding around 200 ms, which is approximately 80 ms later than the peak found for the fixed-rotation object decoding. The time-course of rotation-tolerant object information fits with that found for object decoding when training and testing on different exemplars of the same object in previous work [3–6]. A similar time-course has been found for category coding, which also shows a peak in decoding around 200 ms after stimulus onset [3–6]. This suggests that around this time a rich object representation has been established that is tolerant to rotation and generalises across different exemplars of the same object and category.

The lower decoding accuracy for stimuli presented at the faster compared to slower rate could happen in part due to masking degrading the signal quality generally, and in part due to the fast presentation speed reducing the depth of processing [21,23] and therefore limiting the amount of rotation-tolerant object information. This suggests that recurrent processing, which is expected to be disrupted in the 20 Hz condition, is needed for the formation of rotation-tolerant representations [18]. The reduced rotation-tolerant information for the 20 Hz compared to 5 Hz condition is unlikely to be driven by attentional modulation, as the objects were task-irrelevant and attention-related effects on decoding typically emerge later, after ~200 ms [25,28,49].

The time-course of decoding shows that the object representation persists after the presentation of the next stimulus in the sequence. If one assumes an afferent latency of around 50–70 ms to reach visual cortex [50,51], in line with when substantially above-chance decoding seems to emerge, then 200 ms later in the 5 Hz condition and 50 ms later in the 20 Hz condition, the next stimulus presented will have reached visual cortex, presumably interrupting any feedback loops [52]. However, substantially above-chance decoding persists well beyond these times, which could be explained by a combination of the new stimulus not yet reaching particularly high-level representations and possibly multiplexing, wherein successive stimuli are represented by distinct activation patterns that can coexist [53,54]. Our results add to the previous literature by showing that not only low-level features can persist, but that high-level rotation-tolerant representations of different stimuli can be present in the brain simultaneously. Further, we showed that in the 20 Hz condition, when processing was interrupted before the rotation-tolerant representation was fully formed (at ~120 ms), multiple object representations were maintained, but considerably less rotation-tolerant information propagated through the system.

The decoding results suggest that the fixed-rotation object decoding method relies more on low-level visual differences between images compared to the rotation-tolerant approach. However, it is still possible that the classifier uses low-level differences for the rotation-tolerant image classification. We therefore used RSA to further minimise the effect of these low-level visual differences. Specifically, we correlated RDMs of the EEG data for the two different presentation rates with a model RDM for the rotation-tolerant object, while partialling out different low-level visual models. For the 5 Hz condition, the peak time of the partial correlation between the EEG and the rotation-tolerant model was ~ 184 ms after stimulus onset. This peak time is a similar time as the peak of rotation-tolerant decoding, suggesting higher-level object information contributed more strongly to the rotation-tolerant object decoding than low-level differences between images, as partialling out the low-level visual models did not impact the peak decoding time.

The cross-decoding indicates that the rotation-tolerant object representations for the fast and slow presentation rates are similar, although the asymmetry of some off-diagonal decoding suggests that the representation of rotation-tolerant object information lasted longer for the 5 Hz compared to the 20 Hz condition. This finding is in line with previous work [6,22] that showed a disruption, but not elimination, of object and category processing when objects were presented at 20 Hz compared to 5 Hz. Together, these results suggest that although the faster presentation speed affects object processing, it does not fully disrupt the object processing.

One interesting aspect of the apparent transition from orientation-specific to rotation-tolerant representation is that there appears to be two distinct processing stages rather than a smooth transition, though we are limited by EEG’s sensitivity to cortical responses strongest at the scalp. That is, one might have expected a gradual, smooth decrease in the fixed-rotation information as the rotation-tolerant information emerged. Instead, the data show two peaks in the fixed-rotation object decoding, as if two distinct processing stages are involved. This was also observed in some previous papers [6,20,22]. An interesting aspect of the temporal generalisation data is also consistent with the theory that there are two discrete processing stages. At about the same time as the decoding accuracy peaks, the temporal generalisation matrix shows two peaks of generalisation, which are separated by a short low generalisation interval at about 160 ms. By “low generalisation interval”, we mean that training on the EEG data at that time yields reasonable decoding accuracy but very little generalisation to other times, compared for instance to 220 ms, when the decoding accuracy is about the same but much more generalisation occurs. This is consistent with the brain’s representations at 160 ms being quite transient (not generalising to other times), a sort of transitional period as the rotation-tolerant information emerges. Perhaps the calculations required for the rotation-tolerant information require an intermediate representation or code that is almost immediately discarded.

Taken together, this study provides insight into the temporal dynamics of the emergence of rotation-tolerant object information in the brain. The time-course of rotation-tolerant object representations shows a peak around 200 ms for both the RSA and decoding analyses. This suggests that around this time the object information has been ‘untangled’ [1], and a rich object representation has been established that is tolerant to rotation and generalises across different exemplars of the same object. These results highlight the importance of looking beyond the simple object decoding methods, which cannot separate the contribution of low-level feature processing from object representations that are tolerant to rotations.
